# Connecting Proline and γ-Aminobutyric Acid in Stressed Plants through Non-Enzymatic Reactions

**DOI:** 10.1371/journal.pone.0115349

**Published:** 2015-03-16

**Authors:** Santiago Signorelli, Pablo D. Dans, E. Laura Coitiño, Omar Borsani, Jorge Monza

**Affiliations:** 1 Laboratorio de Bioquímica, Departamento de Biología Vegetal, Facultad de Agronomía, Universidad de la República, Montevideo, Uruguay; 2 Joint BSC CRG IRB Research Program in Computational Biology, Institute for Research in Biomedicine (IRB Barcelona), Barcelona, Spain; 3 Laboratorio de Química Teórica y Computacional (LQTC), Instituto de Química Biológica, Facultad de Ciencias, Universidad de la República, Montevideo, Uruguay; University of Tasmania, AUSTRALIA

## Abstract

The accumulation of proline (Pro) in plants exposed to biotic/abiotic stress is a well-documented and conserved response in most vegetal species. Stress conditions induce the overproduction of reactive oxygen species which can lead to cellular damage. In vitro assays have shown that enzyme inactivation by hydroxyl radicals (^·^OH) can be avoided in presence of Pro, suggesting that this amino acid could act as an ^·^OH scavenger. We applied Density Functional Theory coupled with a polarizable continuum model to elucidate how Pro reacts with ^·^OH. In this work we suggest that Pro reacts favourably with ^·^OH by H–abstraction on the amine group. This reaction produces the spontaneous decarboxylation of Pro leading to the formation of pyrrolidin-1-yl. In turn, pyrrolidin-1-yl can easily be converted to Δ^1^-pyrroline, the substrate of the enzyme Δ^1^-pyrroline dehydrogenase, which produces γ-aminobutyric acid (GABA). GABA and Pro are frequently accumulated in stressed plants and several protective roles have been assigned to these molecules. Thereby we present an alternative non-enzymatic way to synthetize GABA under oxidative stress. Finally this work sheds light on a new beneficial role of Pro accumulation in the maintenance of photosynthetic activity.

## Introduction

When plants are exposed to biotic/abiotic stress, damage on cellular components (proteins, lipids, carbohydrates, and DNA) increase as a result of the overproduction of reactive oxygen species (ROS) such as hydrogen peroxide (H_2_O_2_), singlet oxygen (^1^O_2_), superoxide anion (O_2_·^−^), and hydroxyl radicals (^·^OH) [1]. The latter is the most reactive species among ROS and can be generated *in vivo* either by Fenton’s reaction, in the Haber-Weiss cycle, or through homolysis of H_2_O_2_ under UV radiation to which plants are highly exposed [2,3]. Despite its short lifetime, the production of ^·^OH has been detected in intact plants using EPR techniques [4,5].

Hydrogen abstraction, addition, and electron transfer processes are the most common reaction channels for ^·^OH, leading to new radicals or closed shell molecular species with lower reactivity [6].

Cellular defense against ROS can benefit from either enzymatic or non-enzymatic antioxidant processes. Proline (Pro) has been considered to be involved in the non-enzymatic antioxidant plant defense [7,8]. Accumulation of Pro in stressed plants, up to 100 times the normal level, has been a well-known fact for more than 40 years [9]. In this condition, Pro can reach a cytosol concentrations of 120 to 230 mM (see reference [10] and references therein). Under drought and high salinity conditions, UV/Vis irradiation, oxidative stress, the presence of heavy metals, or as a response to different kind of biotic stresses [11], Pro accumulation by *de novo* synthesis has been reported to be a feature shared by a wide variety of organisms including bacteria, fungi and plants [12].

Early in 1989, Sminorff and Cumbes showed that enzyme inactivation by ^·^OH can be avoided *in vitro* by the presence of Pro, proposing that this molecule might act as a ^·^OH scavenger [13]. Later, it has also been suggested that Pro could protect plants from ^1^O_2_ oxidation [14,15]. Indirect evidence of such protective roles, emerged comparing oxidative damage on genetically-engineered plants under stress, where transgenes were used to control the production of Pro. Under saline conditions, transgenic plants with increased production and accumulation of Pro were less affected by oxidative damage [16], while plants genetically-unable to produce Pro exhibited a significantly lower tolerance to stress [17]. Recently, direct evidences showed that Pro does not quench singlet oxygen (^1^O_2_), as it was thought during several years, concluding that the protective role of Pro against oxidative damage, observed in several plants, could be related to the ^·^OH scavenger activity [18].

A pioneer work of Amici *et al*. suggested that ^·^OH reacts with Pro forming 5-hydroxyproline (5-Hyp), and with 5-Hyp to finally produce Glutamic acid [19,20]. In a recent work, we explored the reactions involving ^·^OH attack to the different C atoms of Pro to evaluate the formation of 5-Hyp, using a theoretical approach, and suggested that the formation of 5-Hyp is unlikely to occur [21]. We predicted that the reaction should always occur on the carboxylate face (*s-face*) of Pro, to produce either 3,4-Δ-Pro or pyrroline-5-carboxylate (P5C) [21].

In this scenario, the present work aims to evaluate the ^•^OH-attack on the N atom to assess the competitiveness of this pathway as opposed to those described on the C atoms [21]. We found out that ^•^OH-attack on the N atom of Pro is competitive with the most favored ones over the C atoms, and can lead to the formation of γ-aminobutyric acid (GABA), which was also reported to accumulate in response to abiotic and biotic stress [22].

## Methods

### Molecular systems

**Fig 1:**
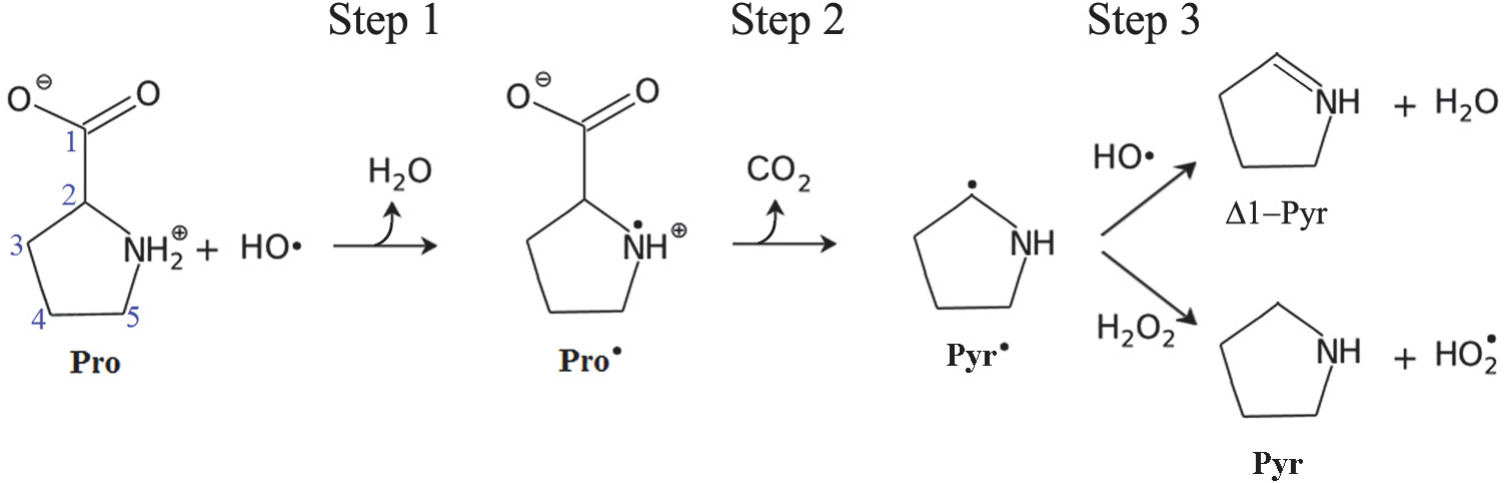
Reactions pathways of Pro zwitterion with ^•^OH in aqueous solution. Atom numbering scheme is shown over Pro in blue numbers.

We explored H-atom abstractions by ^•^OH from the 4-endo and 5-endo conformer of the zwitterionic form of Pro in aqueous solution. In the 4-endo conformation the C2, C3, C5 and N atoms define almost a plane, while in the 5-endo conformer the C2, C3, C4 and N atoms are almost co-planer. As shown in the Fig. 1, the primary step in the reaction of ^•^OH with Pro (Step 1) is characterized by the H-atom abstraction by radical attack to the N atom at both faces of the pyrrolidine ring (i.e. from the side of the carboxylate group or from the opposite one, respectively labeled s/o faces). At Step 2 the release of the carboxylic group as CO_2_ leads to the formation of the radical pyrrolidin-1-yl (Pyr^•^). The Pyr^•^ can react again with ^•^OH -or with other molecules able to abstract hydrogen atom from NH group- to yield delta-1-pyrroline (Δ^1^-Pyr) and water. Alternatively the Pyr^•^ can react with H_2_O_2_ molecule and produce two closed shell molecules: Pyrrolidine (Pyr), and the acid form of superoxide.

### Level of theory

The structures of each stable species (reactants, products, pre-reactive intermediate complexes (IC), and transition states (TS)) were fully optimized in aqueous solution at the (U)M06/6-31G(d,p) level [23] coupled with the IEF-PCM polarizable continuum model [24–26]. Calculations were performed without imposing symmetry restrictions and using solute cavities adapted to the molecular shape and built with Bondi *radii* [27]. An ultrafine pruned grid, having 99 radial shells and 590 angular points per shell was employed for numerical integration in all M06 calculations in order to reduce possible errors in calculating energies and barriers [28]. Expectation values of the spin operator S^2^ were checked to be below 0.76 and 2.01 for doublet and triplet open-shell species, respectively, to minimize spin contamination. The nature of each stationary point was carefully verified by inspection of the eigenvalues of the analytic Hessian in aqueous solution. Thermochemistry was evaluated at 298.15 K relying on the standard treatment for assessing thermal contributions (rigid rotor, harmonic vibrations with no scale factor, etc.) as implemented in the Gaussian09 program [29]. Non-electrostatic contributions (cavitation, dispersion and repulsion) [30,31] to the solvent free energy were also evaluated at 298.15 K. The reaction coordinate of each transition state (TS) was visually inspected by animation of the eigenvector associated to the imaginary frequency. IRC minimum energy reaction paths [32] towards reactants or products were generated with the HPC algorithm [33] including 45 steps for each side with a step size of 2 Bohr/amu^1/2^. All the representative structures obtained from each side of the reaction path, were thus used as the starting point for optimizing the structure of the corresponding intermediate complexes (IC).

The Natural Bond Orbital (NBO) [34,35] population analysis was performed to evaluate the bonding characteristics between the C_1_-C_2_ atoms of reactants, TS, I_C_ and products. The orthogonal set of localized orbitals obtained through this analysis were also used to compute the Wiberg bond index for the decarboxylation process [36,37].

Single-point calculations at the MP2 frozen core level (using the 6-31G(d,p) basis set) were performed to analyze the spin and electron densities of all the characterized structures. The Atoms in Molecules (AIM) approach [38] was used to follow the spontaneous decarboxylation process, finding the bond critical points between the atoms in the plane of the carboxyl group in Pro.

All the calculations were performed using Gaussian09, rev. A.1 or B.1 [29], while the molecular drawings were built using either Gaussview 5 or VMD 1.9.1 [39]. The AIM analysis was achieved with the AIM-UC program [40].

## Results and Discussion

### The amine group of Pro is one of the most favored reaction sites for H-abstraction by the hydroxyl radical

**Fig 2:**
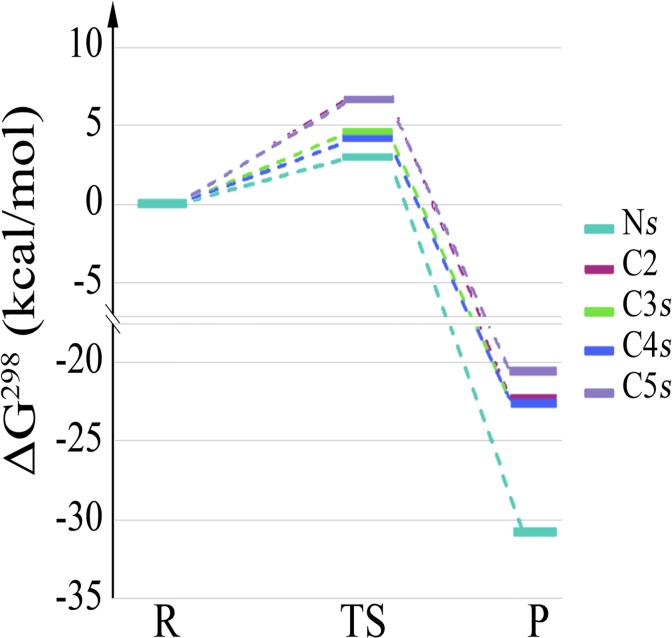
Energy profiles for the most favorable reactions according to Table 1. Free energies (in kcal mol^−1^) relative to the reactants were computed in aqueous solution at 298.15 K. Energies for the H-abstraction at the C atoms were taken from [21].

**Fig 3:**
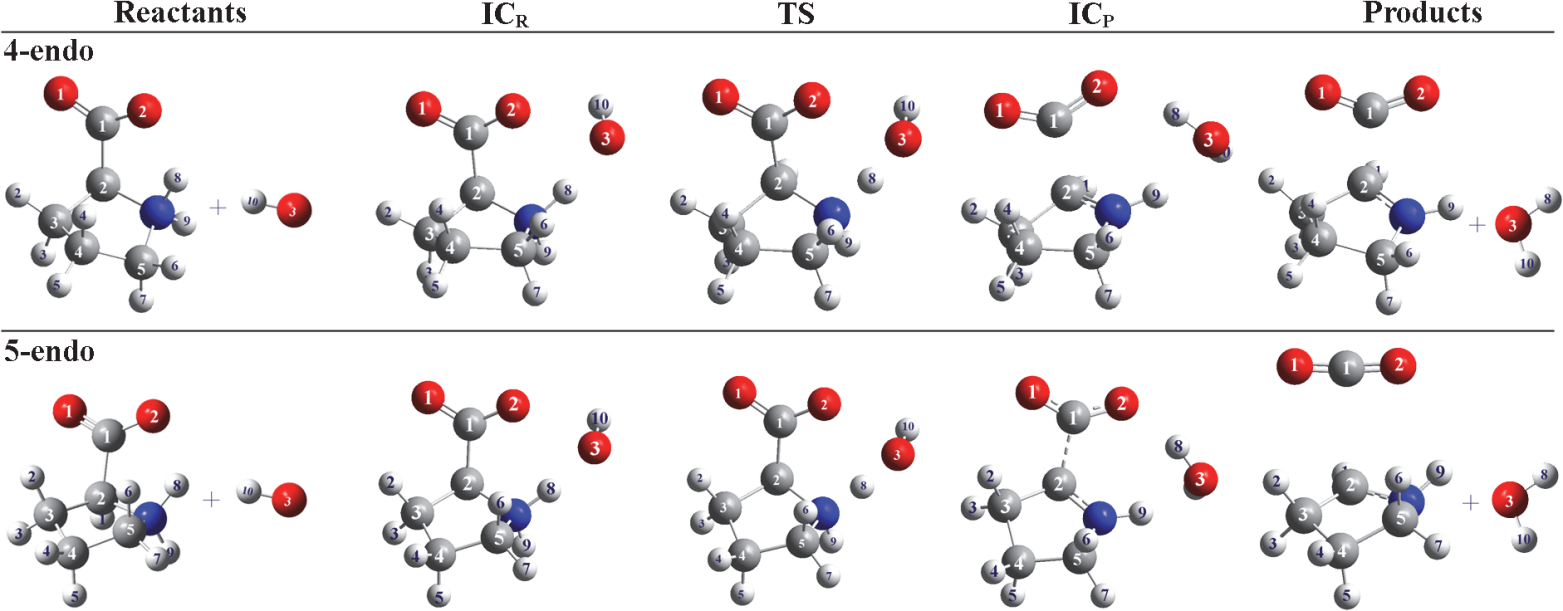
Structures involved in the H-abstraction by ^•^OH from N atom of the 4-endo and 5-endo-Pro. Note that IC_R_ and IC_P_ are the intermediary complexes for the reactants and products side respectively.

Four different reaction pathways for the ^•^OH-attack to the N atom of Pro zwitterion were evaluated. These pathways include, for both 4-endo and 5-endo conformers of Pro, the attack either by the side of the carboxyl group (*s-face*), or its opposite (*o-face*). The corresponding energetic barriers (Table 1) evaluate the competitiveness of these pathways as opposed to those recently described by us [21] on the C atoms. The lowest barriers in terms of free energies for each site of attack are represented in Fig. 2. Remarkably, after the H-abstraction occurs on the N atom of 5-endo Pro, the carboxyl group is destabilized and dissociated from the ring (Fig. 3). The barrier of these reactions are small, lowering when the attack occurs from the *s-face*. Moreover, the barriers related to the *s-face* abstraction over N are lower ∼by 1 kcal/mol than those previously described for the most favorable H-abstractions on C3/C4 atoms (Table 1), all of them near to the diffusion-controlled limit. Pre-reactive complexes, obtained from the ends of the intrinsic reaction coordinate path, are found to be stable in terms of enthalpy (6–7 and 2–3 kcal mol^−1^ for the *s*- and *o*-face respectively). However in terms of free energy these pre-reactive complexes turn unstable due to unfavorable entropic contributions from 2 to 6 kcal mol^−1^. These results would eventually preclude the possibility of these intermediating complexes to act as kinetic traps, as it was found for other H-abstraction by ^•^OH from Leucine dipeptide [41].

**Table 1:** Relative energies (in kcal mol^−1^) for the species involved in Steps 1 in terms of enthalpies (ΔH) and free energies (ΔG) in aqueous solution at 298.15 K.

Site/face	Unimolecular barrier		Bimolecular barrier		Reaction energy	
	ΔH	ΔG	ΔH	ΔG	ΔH	ΔG
Ns 4-endo	−0.30	−0.18	−6.4	3.0	−28.2	−30.8
No 4-endo	3.0	3.9	0.58	8.9		
Ns 5-endo	−0.30	0.73	−7.0	3.1	−34.1	−32.0
No 5-endo	3.3	2.7	−0.09	8.7		
Reference values for C atoms taken from reference [21]						
C2 4-endo	1.1	1.2	−1.5	6.9	−20.1	−22.3
C3s 4-endo	1.7	2.0	−5.1	4.6	−20.1	−22.6
C3o 4-endo	−0.9	−0.3	−3.0	6.2		
C4s 4-endo	1.6	1.5	−5.1	4.1	−20.1	−22.8
C4o 4-endo	1.0	2.3	0.0	8.9		
C5s 4-endo	3.9	5.2	−3.3	6.7	−18.5	−20.7
C5o 4-endo	1.3	0.9	−0.9	7.3		

### Hydroxyl attack at the N atom triggers Pro decarboxylation

Taking into account that the 4-endo and 5-endo forms of Pro exist in solution in equal proportions [42], and that the lower energy barriers were found for those species when the attack occurs on the *s*-face, we focused on the s-face reaction pathway for both species to evaluate in detail the structural changes that produce the release of carboxyl group from the pyrrolidine ring. Fig. 3 shows the structure of reactants, intermediate complexes, transition state and products for this reaction.

Table 2 shows the main structural parameters of the species involved in the Step 1 on *s-face*. Once the abstraction reaction has reached the TS, the distance between the C_α_ and the carboxyl group (C_α_-COO^−^) increases from 1.529 (TS) to 1.795 Å (Product), in the 4-endo conformation, and from 1.528 (TS) to 2.711 Å (Product) in the case of the 5-endo conformer. This reveals a weakening of the C_1_-C_2_ bond, while the shortening of the C_2_-N distance, in both conformations, is characteristic of a double bond (Table 2). On the other hand, the angle O_1_-C_1_-O_2_ of the carboxyl group becomes less acute (particularly in the 5-endo conformer) and the C = O distances of these groups become shorter, resembling to the CO_2_ molecule. When the abstraction occurred from 5-endo Pro no minima was found on the product side until a complete dissociation of the carboxyl group occurs (the final C_1_-C_2_ distance is 2.711 Å, and the value of the O_1_-C_1_-O_2_ angle is 173.4°). These results provide strong evidence that the ^•^OH-attack on the N site of Pro might trigger its decarboxylation, giving the formation of pyrrolidin-1-yl (Pyr^•^). The Pro decarboxylation has also been evidenced experimentally *in vitro* in presence of oxidative species, such as permanganate [43] and copper [44]. In the same way, Bonifacic *et*. *al*. [45] studied the reaction between ^•^OH and Gly finding that ^•^OH attacks occur exclusively on the NH_2_ group, leading to the formation of HN^•^-CH_2_-CO_2_^−^ and ^+^H_2_N^•^-CH_2_-CO_2_^−^. The latter compound decompose (on the nanosecond time scale) to CO_2_ and H_2_N-^•^CH_2_ [45].

**Table 2:** Selection of main structural parameters involved in Steps 1.

	4-endo conformation					5-endo conformation				
	Reactive	IC_R_	TS	IC_P_	Product	Reactive	IC_R_	TS	IC_P_	Product
*Distances (angstroms)*										
C_1_-C_2_	1.546	1.536	1.529	**1.722** ^**a**^	**1.795**	1.543	1.535	1.528	**1.704**	**2.711**
C_2_-N	1.510	1.494	1.481	**1.382**	1.373	1.505	1.495	1.482	**1.384**	1.397
N-H_8_	1.045	1.051	**1.207**	**2.709**	-	1.040	1.052	**1.202**	**2.696**	-
C_1_-O_1_	1.243	1.241	**1.235**	1.222	1.219	1.243	1.241	**1.236**	**1.223**	**1.168**
C_1_-O_2_	1.258	1.267	1.281	**1.231**	1.218	1.258	1.267	1.280	**1.233**	**1.168**
O_2_-H_8_	1.808	**2.329**	**2.287**	**1.825**	-	1.847	**2.186**	**2.284**	**1.786**	-
O_3_-H_8_	-	1.670	**1.278**	**0.978**	-	-	1.668	**1.285**	**0.980**	-
*Angles (degrees)*										
C_2_-C_1_-O_2_	115.8	**119.2**	120.3	**112.2**	109.4	115.6	**119.6**	120.6	**113.5**	**92.1**
O_1_-C_1_-O_2_	128.9	125.5	122.0	**135.4**	**139.9**	128.9	125.3	122.0	**134.6**	**173.2**
N-C_2_-H_1_	109.4	109.0	109.5	**113.4**	115.2	108.1	108.0	108.7	112.4	**119.1**
N-H_8_-O_3_	-	163.4	**168.4**	**85.6**	-	-	162.1	**168.0**	**86.5**	-
*Dihedral (degrees)*										
H_8_-N-C_1_-O_2_	2.7	**26.4**	**19.1**	**5.1**	-	−8.7	**23.9**	**12.4**	8.7	-

^a^ Note that major changes are highlighted in bold.

To further characterize steps 1 and 2 (see Fig. 1), the H-abstraction and the *a posteriori* decarboxylation were analyzed using the Wiberg indices, spin densities (the difference between the alpha and beta densities) and the AIM approach.

In Table 3 we present the Wiberg index for the C_1_-C_2_ bond in both 4-endo and 5-endo conformations along the structures of step 1. As shown, the results are in agreement with the geometrical parameters (Table 2). The C_1_-C_2_ bond is slightly strengthened up to the TS, and then quickly weakens being almost completely broken in the product of the 5-endo. In both conformers we exhaustively search for a TS on the decarboxylation process, but all the attempts led to a quick release of the CO_2_ group, suggesting that step 2 is barrierless.

**Table 3:** Wiberg bond index for the C_1_-C_2_ bond of the 4-endo and 5-endo conformations of proline along the step 1.

	C_1_-C_2_ bond in 4-endo conformation				
	Reactive	IC_R_	TS	IC_P_	Product
Wiberg bond index	0.9117	0.9245	0.9379	0.6385	0.5529
% relative to reactive	100	101	103	70	**61**
	C_1_-C_2_ bond in 5-endo conformation				
	Reactive	IC_R_	TS	IC_P_	Product
Wiberg bond index	0.9147	0.9292	0.9434	0.6657	0.0441
% relative to reactive	100	102	103	73	**5**

**Fig 4:**
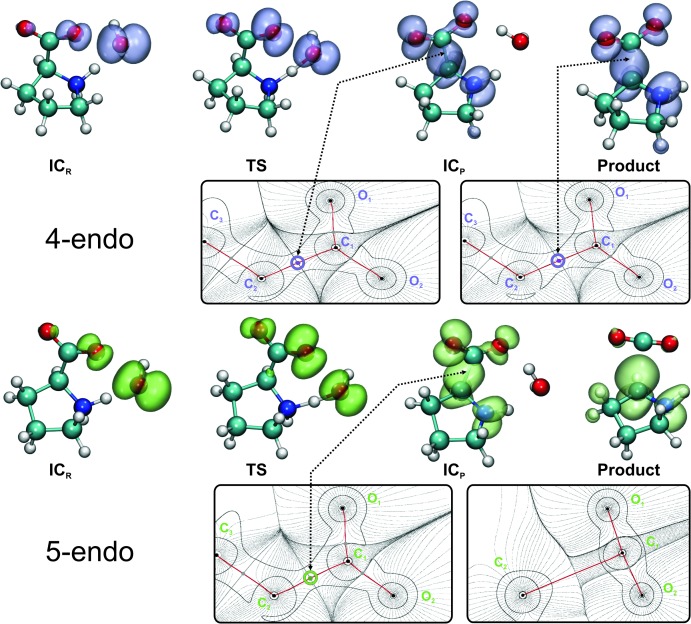
Spin densities and AIM analysis. For the sake of comparison, the same isosurface of value 0.003 |e|/Å^3^ representing the difference between the alpha and beta electron densities was depicted in violet or green, for the 4-endo and 5-endo forms respectively. For the products and the intermediate species on the product side (IC_P_), the presence of bond critical points (bcp) was evidenced by means of AIM. The bcp connecting the carboxyl group to the pyrrolidine ring is depicted in violet or green, whereas the remainder bcps are shown in gray. The nuclear critical points (located at the position of the nuclei), the basin paths, and the gradient field are depicted in solid or dashed black lines. The bond paths, defined by the chosen 2D projection (plane), are shown in red.

The spin density is a good quantum descriptor to study the unpaired electron along the reaction path. As shown in Fig. 4, the unpaired density moves from being delocalized over the oxygen atoms (see IC_R_ and TS in Fig. 4) to be localized over the C_2_/Cα and N atoms in the products (see IC_P_ and Products). During the H-abstraction, the spin density moves from the oxygen atoms (mainly from ^•^OH), through the N atom (forming a cationic radical), to the C_2_/Cα atom, leading to the decarboxylation of the Pro and the strengthening of the Cα-N bond (see Table 2). The final result is the spontaneous formation of a Pyr^•^ and carbon dioxide (clear in the case of the 5-endo conformer), in a barrierless reaction, for which no activation energy is required and no TS can be defined. It is worth mentioning that the decarboxylation occurs differently in the 4-endo and 5-endo forms. The decarboxylation seems to be well defined in the 5-endo. In the 4-endo reaction the spin density does not show a significant differences between IC_P_ and the product (Fig. 4), although, as mentioned before, the C_1_-C_2_ distance slightly increases between these two stable species (see Table 2), suggesting that we found a stable complex, in the product side of the H-abstraction, before decarboxylation (in agreement with the Wiberg indices reported in Table 3). In the latter case, also the AIM analysis reveals the presence of a bond critical point (bcp), suggesting that there still is a covalent bond connecting the carboxyl group to the pyrrolidine ring. This bcp is not present in the product of the 5-endo reaction where the decarboxylation is evident. In the 4-endo form, the Pyr^•^ and the CO_2_ molecules seem to be trapped in a local minimum forming a stable complex, which could explain in part the low rate of decomposition observed experimentally [45].

### Complementary reactions to obtain closed-shell species

**Fig 5:**
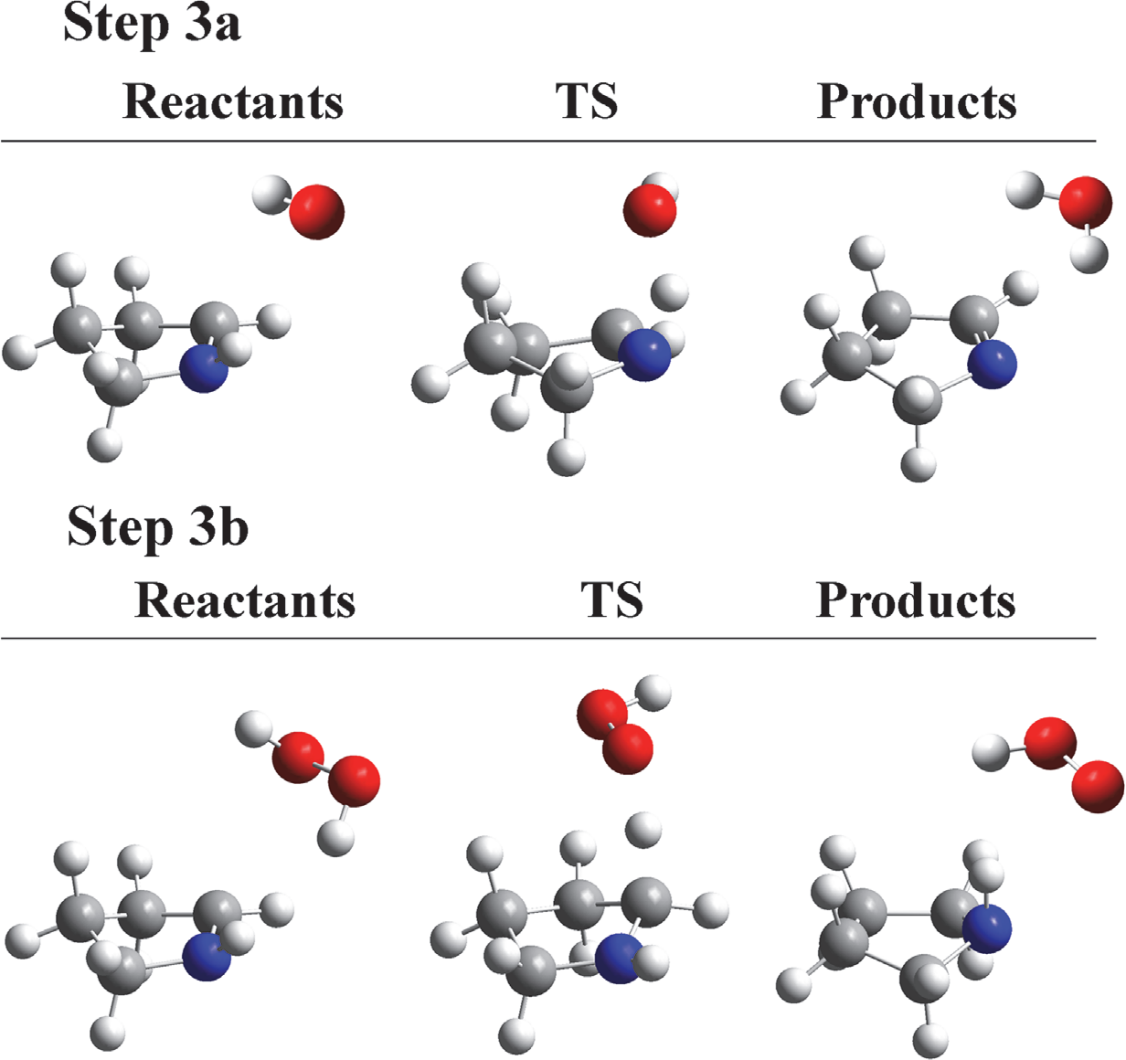
Structures involved in Step 3. In Step 3a, the H-abstraction from Pyr^•^ at the N atom by ^•^OH produces Δ^1^-Pyr. In Step 3b, the H-abstraction from H_2_O_2_ by Pyr^•^ produces Pyr.

The Pyr^•^ can react with different molecules to produce closed shell molecules. Here we suggest two different mechanisms: one involves another H-abstraction by ^•^OH from the N atom of the pyrrolidine ring; and the other one involves the H-abstraction by Pyr^•^ from hydrogen peroxide (see Fig. 1). In both cases the reaction leads to the formation of Δ^1^-Pyrroline (Δ^1^-Pyr). Note that after decarboxylation it is not possible to define the s- and o-face, and for this reason there is only one possible conformation in the two reactions explored in Step 3 (Fig. 5).

As expected (in Step 3) the reaction with ^•^OH has a lower energy barrier as well as being more exothermic than the reaction with H_2_O_2_ (see Table 4). Both proposed mechanisms are likely to occur, and while Step 3a is irreversible, Step 3b can occur in both directions. Once again the intermediate complexes are more stable in terms of enthalpies, but not anymore when entropic contributions are considered.

**Table 4:** Relative energies (in kcal mol^−1^) for the species involved in Steps 3 in terms of enthalpies (ΔH) and free energies (ΔG) in aqueous solution at 298.15 K.

Step	Site of attack	Attacked by	Unimolecular barrier		Bimolecular Barrier		Reaction Energy	
			ΔH	ΔG	ΔH	ΔG	ΔH	ΔG
3a	N	^•^OH	4.4	5.1	−1.8	8.1	-88.7	-88.7
3b	C_2_	H_2_O_2_	5.4	6.6	−6.4	11.7	-5.1	-4.2

### Connecting Pro to GABA

**Fig 6:**
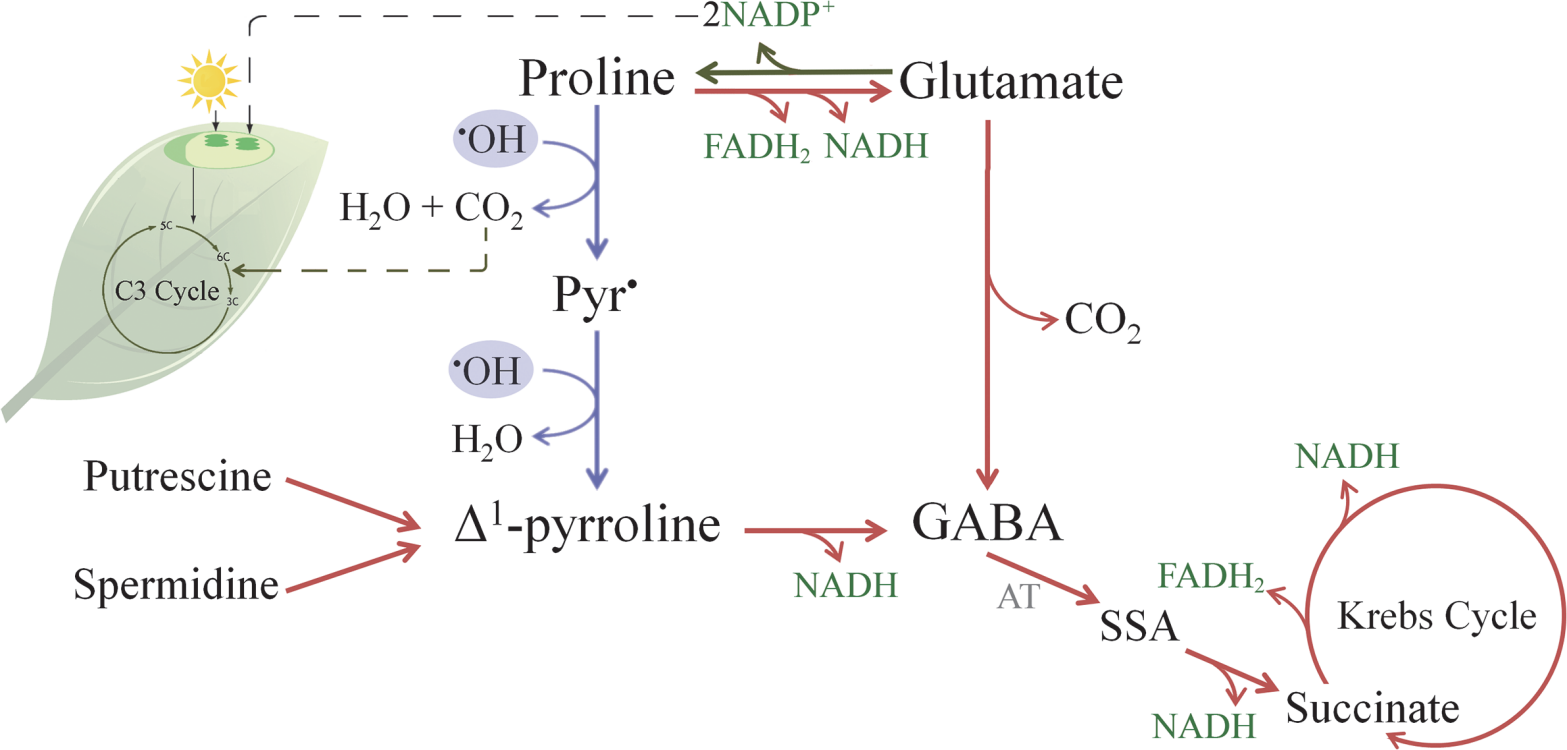
Suggested pathway to connect Pro and GABA and its possible implications in major metabolic processes. Blue lines represent the non-enzymatic reactions proposed to connect Pro and Δ^1^-Pyr by ^•^OH scavenging. In the non-enzymatic reactions two ^•^OH are captured and the CO_2_ released could enter the C3 cycle. Red and green lines represent catabolic and anabolic pathways respectively. SSA stands for succinic semialdehyde, and AT stands for amino transferases.

In plants, Δ^1^-Pyr produced in Step 3a is the substrate of pyrroline dehydrogenase (PYRR-DH) which converts it into γ-aminobutyric acid (GABA) [46]. GABA is well-documented to accumulate under stress conditions [47]. For this reason, several protective roles had been assigned to GABA, such as the contribution to the C:N balance, the regulation of cytosolic pH, the protection against oxidative stress, the defense against insects, the osmoregulation, and cell signaling [47]. Most of these protective roles have also been attributed to Pro [11]. The principal way in which GABA is synthetized is from glutamic acid, by the enzyme glutamate decarboxylase [22], while a secondary pathway involves polyamines and the formation of Δ^1^-Pyr [46,48]. For example, the CMSII mutant of *Nicotiana sylvestris* has reduced glutamate decarboxylase (GAD) activity, but the GABA content nevertheless increased more than two- fold from the base level when treated with NaCl [49], suggesting that the accumulation of GABA could be mediated by polyamines oxidation. This pathway requires the activity of pyrroline (γ-aminobutyraldehyde) dehydrogenase, an enzyme not produced in stress conditions [48], meaning that GABA formation should be mediated by the accumulation of GABA precursors. Here we propose an alternative non-enzymatic pathway (shown in blue in Fig. 6) in which Pro can contribute to the formation of the GABA precursor Δ^1^-Pyr, giving a possible explanation to connect the simultaneous accumulation of Pro and GABA that occur in stress conditions. We recently demonstrated that a plastidic glutamine synthetase mutant of *Lotus japonicus* with unaltered glutamate content but lower proline content, had a lower accumulation of GABA in response to osmotic stress when compared with the wt [50].

### Biological significance of the reaction of Pro with ^•^OH in plants under stress

In the chloroplasts, the co-localization of Pro, ^•^OH and H_2_O_2_ is essential for the proceeding of the reactions described in Steps 1 and 3. In these organelles, the electron leakage of O_2_ is the major source of O_2_^•−^ [51], which leads to the formation of H_2_O_2_ by SOD activity in a diffusion-limited reaction. ^•^OH can be produced from H_2_O_2_ by Fenton’s reaction or by homolytic cleavage under UV exposure. In addition, Pro reaches concentrations of at least 160 mM [52] (even in non-stressful condition) into these organelles becoming more abundant than every common antioxidant (i.e.: 0.8–2.4 mM for glutathione; 12–25 mM for ascorbate) [53].

Besides the H_2_O_2_ production in chloroplasts, H_2_O_2_ is also generated by photorespiration in the peroxisomes [54]. Other H_2_O_2_ sources are the copper-containing oxidases and peroxidases, being the latter the main responsible for H_2_O_2_ production during oxidative burst in stress conditions in plants [54]. Since H_2_O_2_ has a long lifetime and can diffuse through organelles [55], it can be assumed to be available in all the cell to yield ^•^OH or react with Pyr^•^.

Because of the main co-localization of ROS and Pro in chloroplasts, the CO_2_ released from Pro accumulates predominantly in these organelles. There, the photosynthetic machinery is able to use CO_2_ by fixation through the C3 Cycle (see Fig. 6). Despite being a hypothesis, that will require further experimental confirmations, this localized production of CO_2_ could be an advantage for plants under stress conditions (i.e. drought). In such conditions the stomata are close to avoid water loss, limiting the uptake of CO_2_, that leads to a reduction in carbon fixation and the accumulation of reducing power (NADPH). All these combined features will most probably produce ROS by electron leakage. Therefore, it is not unreasonable to think that the CO_2_ produced during Pro reactions would help reducing the accumulation of NADPH and the generation of ROS.

Pro synthesis in green tissues was proposed to regenerate NADP^+^, helping in the maintenance of an adequate NADP^+^/NADPH ratio inside the cells [56], and that Pro could translocate to the roots to be catabolized [56]. In addition to the NADPH consumption produced during Pro synthesis, our work suggests that Pro could also help to reduce the NADP^+^/NADPH ratio, by releasing CO_2_ that will enter the C3 cycle and consume NADPH.

On the other hand, it is known that transaminases turn GABA into succinic semialdehyde (SSA) that is then converted to succinate by succinic semialdehyde dehydrogenases (Fig. 6) [57,58]. These reactions, known as GABA shunts, produce substrates of the mitochondrial respiratory chain (succinate and NADH), which ultimately generates ATP [47]. GABA shunts affect the redox balance in the cell, because succinate enters the Krebs Cycle bypassing three sites of NADH production, thus reducing the NADH/NAD^+^ ratio [49,59,60]. This reduction in NADH/NAD^+^ ratio activates key enzymes of the Krebs cycle [61]. Additionally, it is known that the succinyl-CoA ligase and the α-ketoglutarate dehydrogenase enzymes are degraded under oxidative stress, limiting the ATP production in the Krebs cycle [62]. GABA shunts assure the production of ATP even in stressed conditions, reason why GABA shunts were considered a protection against oxidative stress [47].

Overall, we consider that the non-enzymatic connection between Pro and GABA presented in this work is a reasonable alternative to catabolize Pro obtaining reducing power, ATP, carbon and nitrogen, even in stressed conditions when the catabolic enzymes of Pro are inactivated. This beneficial aspects of Pro and the protective role against ^•^OH are in line with the multifunctional role that has been assumed for Pro.

## Conclusions

Hydrogen abstraction from the N atom of Pro by ^•^OH radical produces the decarboxylation of Pro, and leads to the formation of Pyr^•^. This reaction mechanism, proposed herein for the first time, is competitive as opposed to those described previously for the C atoms [21]. These theoretical results are in line with experimental data about amino acid decarboxylation under ^•^OH attacks. The Δ^1^-Pyr produced by H-abstraction from Pyr^•^ would contribute to the production of GABA under PYRR-DH catalysis, an essential molecule for plants, that also accumulates in stress conditions. Thereby, we proposed an alternative way to synthetize GABA, through a non-enzymatic reaction relevant in plants under oxidative stress. Finally, this work highlights a new beneficial characteristic of Pro accumulation, as the contribution to maintain the photosynthetic activity in stressed plants.
